# Improvements in Usability of MoCA XpressO, a Self‐administered Digital Cognitive Pre‐screening Tool

**DOI:** 10.1002/alz.086424

**Published:** 2025-01-09

**Authors:** Johanna Gruber, Katie Bodenstein, Richard Jansen, Marion Paquette‐Masson, Joana Krieger, Murray Gillies, Ziad Nasreddine

**Affiliations:** ^1^ MoCA Clinic and Institute, Greenfield Park, QC Canada

## Abstract

**Background:**

XpressO from Montreal Cognitive Assessment (MoCA) is a self‐administered, digital cognitive screening test composed of memory and executive function tasks. Its purpose is to provide a brief pre‐screening of cognition in order to determine whether further cognitive testing is required. A previous validation study comparing the XpressO to the gold standard MoCA test revealed a strong predictive value. However, of the 118 participants recruited, 25% of the participants (n = 30) did not complete the XpressO test due to impaired comprehension (n = 10), requiring assistance (n = 4), skipped or uncompleted tasks (n = 11), and technology difficulties (n = 5). After a qualitative assessment of the users’ experience several improvements were envisioned and implemented in XpressO. This study aims to assess whether these improvements result in a higher completion rate in older adults.

**Method:**

15 older adults that were referred to the MoCA Clinic were recruited to participate in this qualitative study. Each performed the XpressO test while being observed. The observers noted when people experienced difficulty and fed this back to the usability engineer for improvements. As a next step, 30 participants will be recruited from the MoCA Clinic in Greenfield Park, Quebec. Eligibility criteria will include: ability to speak English or French, adults aged 50 years or over, at least 6 years of formal education, and completion of a recent MoCA test within 3 months prior to the start of the study. Participants will complete the XpressO followed by the User Experience Questionnaire (UEQ). The completion rate of XpressO version 1 and version 2 will be compared using a chi‐squared test. To assess whether completion of the XpressO is dependent on MoCA score, an independent t‐test will be used.

**Result:**

Results from the 30 participants will be available by July 2024.

**Conclusion:**

The MoCA XpressO is emerging as an important tool in cognitive pre‐screening and facilitating early detection of cognitive impairment. However, barriers to accessibility need to be addressed in order to optimize the useability of the XpressO for the older population.

